# Comparison of percutaneous balloon compression and microvascular decompression in the treatment of trigeminal neuralgia

**DOI:** 10.12669/pjms.39.5.8049

**Published:** 2023

**Authors:** Zhenyong Li, Zhuqing Xie, Guoqiang Tang, Shihui Jin

**Affiliations:** 1 Zhenyong Li, Department of Neurosurgery, Chenzhou First People’s Hospital, Chenzhou 423000, Hunan Province, P.R. China; 2 Zhuqing Xie, Department of Neurosurgery, Chenzhou First People’s Hospital, Chenzhou 423000, Hunan Province, P.R. China; 3 Guoqiang Tang, Department of Neurosurgery, Chenzhou First People’s Hospital, Chenzhou 423000, Hunan Province, P.R. China; 4 Shihui Jin, Department of Neurosurgery, Chenzhou First People’s Hospital, Chenzhou 423000, Hunan Province, P.R. China

**Keywords:** Masticatory muscle strength, Microvascular decompression, Percutaneous balloon compression, Trigeminal neuralgia

## Abstract

**Objective:**

To compare the effect of percutaneous balloon compression (PBC) and microvascular decompression (MVD) in the treatment of trigeminal neuralgia (TN).

**Methods:**

Data of 98 patients with TN, admitted to Chenzhou First People’s Hospital from May 2020 to May 2022, were retrospectively collected. Patients were divided into two groups based on the surgical method. A total of 53 patients treated with PBC comprised the PBC-group and 45 patients treated with MVD comprised the MVD-group. The immediate pain relief, long-term efficacy, surgical complications, and masticatory muscle strength of the two groups were compared and analyzed.

**Results:**

There was no significant difference in the immediate pain relief and long-term e efficacy, between the two groups (*P*>0.05). Complication rate in the PBC-group was significantly lower than that in the MVD-group (3.77% *vs* 17.78%, *P*<0.05). Medical records within 14 days after the operation showed that the incidence of facial numbness and masticatory muscle weakness in the PBC-group were 37.74% and 28.30% respectively, significantly higher than those in MVD-group (4.44% and 2.22%) (*P*<0.05). These symptoms gradually improved three months after the surgery, and were almost completely resolved after six months.

**Conclusions:**

Compared with MVD, PBC has the same effect in the treatment of TN. PBC is a minimally invasive, safe, and effective method with a low complication rate. Although masticatory muscle strength is slightly impacted by PBC, it gradually recovers within six months after the operation.

## INTRODUCTION

Trigeminal neuralgia (TN) is a common type of cerebral nerve disease with a highly complexed etiology. It is generally believed that TN is related to the compression of blood vessels on the brain stem and the demyelinating area of the trigeminal nerve.^1^ Nerve root compression that results from the arachnoid adhesion or venous contact with the nerve, and the demyelinating area damage led to severe pain attacks in patients.^2^ At early stages, TN is mainly localized in the facial area. However, as the disease progresses, head, back of the ear, shoulder, etc. may be affected, which seriously impacts patient’s quality of life.^3^ While symptoms, associated with mild TN can be controlled with carbamazepine, oxacillin and other drugs, severe symptoms cannot be completely alleviated, which may lead to repeated pain attacks, long-term drug use, and associated adverse drug reactions. For these patients, surgery can be selected.^4^

Currently, surgical methods for TN treatment include microvascular decompression (MVD), percutaneous balloon compression (PBC), and percutaneous radiofrequency radical resection. MVD can relieve pain while avoiding sensory disturbance by cushioning off the nerves and blood vessels. However, MVD involves craniotomy, which is traumatic and is associated with many complications.^5^ PBC is a minimally invasive operation that has become popular in the recent years. It reduces pain by using a balloon to compress the meniscus of the trigeminal nerve, altering the sequence of the meniscus, selectively destroying large myelinated nerve fibers, and promoting demyelination of the trigeminal nerve.^6^ Several studies have investigated the efficacy and postoperative complications of PBC, but the findings are conflicting.^7^,^8^ Therefore, the aim of this study was to further explore the application value of PBC and to compare it with MVD in the treatment of TN patients.

## METHODS

Medical records of 98 patients (49 males and 49 females) with TN who were treated in Chenzhou First People’s Hospital from May 2020 to May 2022 were retrospectively reviewed. The average age of the patients was 56.02 ± 12.01 years. Patients who received PBC (n=53) were set as the PBC-group, and patients who received MVD (n=45) were set as the MVD-group.

### Inclusion criteria:

- Patients met the diagnostic criteria of primary TN.^9^
- Patients with complete medical records.
- Patients aged ≥ 18 years.
- Patients who failed to respond to oral carbamazepine treatment

### Exclusion criteria:

- Patients with secondary TN.
- Patients complicated with other serious organic diseases and malignant tumors.
- Patients with dysfunction of important organs.
- Patients without drug therapy.
- Patients who stopped the treatment and discontinued follow-up

### Ethical Approval:

This study was approved by the Medical Ethics Committee of Chenzhou First People’s Hospital (No. 2020059, Date: 2023-01-31). Informed consent was obtained from all patients.

For the PBC-group, the patient’s posture was adjusted to supine position after the anesthesia, and the head was fixed using the head ring to ensure that both external auditory meatus are at the same level. Hatel puncture approach was selected and a puncture point 2.5cm beside the corner of the mouth was done. A line was drawn 3cm below the pupil of the affected side and in front of the external auditory canal at the zygomatic arch level. Head position was adjusted again under X-ray fluoroscopy. Needle was inserted along the puncture point, direction and angle of the needle were adjusted according to the drawn line, and the round hole was punctured. When the needle tip reached the round hole, the needle core was pulled out, and the balloon was inserted into the round hole through the puncture needle. Then, under X-ray fluoroscopy, the balloon was put into Meckel’s cavity, the balloon guide wire was pulled out, Iohexol was injected into the balloon through a 1ml syringe, the balloon was filled, and after maintaining for three to five minutes, the balloon and puncture needle were emptied. The balloon and puncture needle were removed, the puncture point was compressed to stop the bleeding, and a sterile dressing was applied to complete the operation.

For the MVD-group, the patient’s position was adjusted to the affected side after the anesthesia, and a straight incision was made under the star point behind the left ear to separate the subcutaneous tissue to the occipital bone. The hole was drilled at the star point, and the oval bone flap was milled down with a milling cutter 2.5cm × 3cm, exposing transverse sinus and sigmoid sinus. Mastoid air chamber was blocked with bone wax, dura mater was cut in “Y” or “U” shape, suspended on the skull. Cerebellomedullary cistern was opened, slowly releasing cerebrospinal fluid, reducing cerebral pressure. Responsible vessel was located using the microscope; after confirmation, adhesive arachnoid membrane was separated, and Teflon cotton was placed between trigeminal nerve and responsible vessel. After detailed confirmation, bleeding was stopped, and the bone flap replaced. The skull was closed, and the operation was completed.

### Clinical efficacy was evaluated using the following criteria:

- Immediate pain relief after taking the medicine; Pain, slightly relieved after taking the medicine was considered Grade-IV; Noticeable pain that was not relieve by medication was considered Grade-V.^10^ Immediate pain relief rate after the operation = (Grade-I+Grade-II)/total number of cases; long-term effective rate= (Grade-I+Grade-l II+Grade-III)/total number of cases.
- Surgical complications. The incidence of complications such as angular herpes, tinnitus, intracranial infection, diplopia, headache, and facial swelling of the two groups were counted.
- Masticatory muscle strength. The occurrence of facial numbness and masticatory muscle weakness in the two groups from one to 14 days after the operation were counted to evaluate the masticatory muscle strength of the two groups, and the follow-up records were checked to understand the disappearance time of such symptoms.

### Statistical analysis:

SPSS22.0 was used for data analysis. The normality of the data was evaluated using the Shapiro Wilk test. The data of normal distribution were expressed as mean±standard deviation, and the intergroup comparison was performed by independent sample *t* test. The data of non-normal distribution were expressed as median and interquartile interval, and Mann Whitney U test was used for inter group comparison. Non-grade counting data were analyzed using *χ^2^* and represented as [n (%)]. *P*<0.05 indicated statistically significant difference.

## RESULTS

A total of 98 patients were included in this study, all of whom received surgical treatment. As summarized in Table-I, 53 patients (24 men and 29 women) underwent PBC. The average age of this group was 56.66±11.48 years. The median course of the condition was 3 (2, 3) years. Pain location was as follows: left -15 cases, right- 32 cases, bilateral- six cases. A total of 45 patients (25 males and 20 females) were treated by MVD. The average age of patients was 55.27±12.71 years, and the median TN course was 3 (2, 3) years. Pain location was as follows: left-17 cases, right- 21 cases, bilateral -seven cases. There was no significant difference in the general data between the two groups (*P*>0.05) (Table-I).

**Table-I:** Comparison of General Data of Two Groups.

Group	n	Gender (male/Female)	Age (year)	Course of disease (year)	Pain location (n)		

					Left	Right	Bilateral
PBC-group	53	24/29	56.66±11.48	2(2, 3)	15	32	6
MVD-group	45	25/20	55.27±12.71	3(2, 3)	17	21	7
χ^2^/t/Z	-	1.027	0.570	-0.541	1.844		
P	-	0.311	0.570	0.588	0.398		

Immediate pain relief and long-term effective rates were similar between the two groups (*P*>0.05) (Table-II). The complication rate in the PBC-group was 3.77%, significantly lower than that in the MVD-group (17.78%) (*P*<0.05) (Table-III). The incidence of facial numbness and masticatory muscle weakness within 14 days after the operation in the PBC-group were 37.74% and 28.30%, respectively, significantly higher than 4.44% and 2.22% in the MVD-group (*P*<0.05) (Table-IV). As shown by the follow-up data, these symptoms were gradually alleviated three months after the surgery, and basically disappeared six months after the surgery.

**Table-II:** Comparison of immediate pain relief and long-term effective rate between the two groups [n (%)].

Group	n	Immediate pain relief rate	Long term efficiency
PBC-group	53	52 (98.11)	51 (96.23)
MVD-group	45	43 (95.56)	43 (95.56)
χ^2^	-	0.536	0.028
*P*	-	0.464	0.867

**Table-III:** Comparison of complications between the two groups [n (%)].

Group	n	complication						Total

		Angular herpes	Tinnitus	Intracranial infection	Diplopia	Headache	Facial swelling	
PBC-group	53	1 (1.89)	0 (0.00)	0 (0.00)	1 (1.89)	0 (0.00)	0 (0.00)	2 (3.77)
MVD-group	45	1 (2.22)	1 (2.22)	1 (2.22)	1 (2.22)	2 (4.44)	2 (4.44)	8 (17.78)
χ^2^	-							5.209
P	-							0.022

**Table-IV:** Comparison of masticatory muscle strength between the two groups [n (%)].

Group	n	Facial numbness	Masticatory myasthenia
PBC-group	53	20 (37.74)	15 (28.30)
MVD-group	45	2 (4.44)	1 (2.22)
χ^2^	-	15.493	12.117
P	-	0.000	0.000

## DISCUSSION

Our results showed that PBC has better effect and fewer complications compared to MVD. The mechanism of TN has not been fully clarified. However, general consensus is that the TN symptoms are due to the damage of trigeminal nerve root and trigeminal nucleus that cause demyelinating degeneration and abnormal discharge of nerves.^11^ Therefore, antiepileptic drugs such as carbamazepine can alleviate the symptoms of TN patients to a certain extent. However, this treatment is not effective in some patients, and surgical treatment should be considered.^12^

MVD has been used in clinical practice for more than 50 years, and can achieve an early remission rate of 83%~98%.^13^ However, MVD requires craniotomy that is associated with certain risks such as facial nerve palsy, hearing impairment, paralysis, etc.^14^,^15^ In recent years, the development and advancement of minimally invasive surgical technologies such as PBC, have become increasingly popular in the treatment of TN. PBC is an efficient method that is associated with less trauma and fewer complications.^11^,^12^ The results of our study are similar to those of Ma C et al^14^ suggesting that while both PBC and MVD achieve ideal immediate postoperative pain relief and long-term effective rate in the treatment of TN, PBC is associated with lower rate of surgery-related complications.

Zheng S et al.^7^ retrospectively analyzed 1313 patients who received PBC or radiofrequency thermocoagulation (RFT) for TN and showed that the rate of complications among patients in the PBC group were significantly lower compared to the RFT group. However, a systematic review of 481 PBC patients by Texakalidis P et al.,^8^ which included data from five studies, found that the risk of masticatory weakness after PBC surgery was higher. Similarly, the proportion of masticatory weakness in our study was as high as 28.3%. One explanation for these results may be that the trigeminal nerve motor branch component is a special visceral motor fiber, and the branch reaches the masticatory muscle. During the PBC treatment, puncture may cause damage to the trigeminal nerve motor branch. Furthermore, balloon compression may also damage the trigeminal ganglion, causing facial numbness and reduction of masticatory muscle strength after the surgery.^16^,^17^

In our study, the incidence of facial numbness and masticatory muscle weakness in patients treated with PBC was higher than in patients treated with MVD, which was consistent with the findings of Ni H et al..^18^ However, further follow-up found that these symptoms were significantly reduced about three months after the surgery, and almost completely alleviated six months after the surgery. Our results further confirm safety and efficiency of PBC in the treatment of TN patients. Although complications, such as facial numbness and masticatory muscle weakness may occur, they are tolerated well by most patients and can easily resolve over the time after the surgery.^19^

Based on our results, PBC may be adopted as a preferred method of surgical treatment of TN patients. We suggest that in clinical practice, when treating patients with TN, the patient’s physical condition and tolerance to surgery should be comprehensively assessed, and special precautions should be taken during the three steps of the PBC procedure:

### Positioning of foramen ovale:

When implementing this step, the C-arm X-ray machine should be used flexibly, and the angle should be adjusted continuously to accurately identify the foramen ovale and avoid damaging the surrounding structures.^20^

### Balloon implantation:

The route should be accurately planned, guided by the C-arm, and confirmed that the tip of the catheter enters the junction of the petrous bone ridge at the slope.

### Ganglion compression:

As for the shape of balloon, it should be adjusted promptly if it deviates from the pear shape. The filling volume is recommended to be 0.5~1.0ml,^21^ and the compression time is suggested to be more than ten minutes to obtain better effect. However, it may increase the risk of postoperative facial sensory disturbance.^22^ At present, the compression time in clinical practice is usually one to three minutes.^3^,^23^ Since there are no unified standards in this field in China, PBC parameters can be reasonably adjusted according to clinical practice to find the best balance between the clinical efficacy and complications.

### Limitation of the study:

This was a single-center retrospective study that included only 98 patients and did not have long-term follow-up. There were fewer observation indicators included which may make the conclusions subjective and one-sided.

## CONCLUSION

Compared with MVD, PBC has the same effect in the treatment of TN. PBC is a minimally invasive, safe, and effective method with a low complication rate. Although masticatory muscle strength is slightly affected by the PBC procedure, it gradually recovers within six months after the operation.

### Authors’ contributions:

**ZL:** Conceived and designed the study.

**ZX**, **GT** and **SJ:** Collected the data and performed the analysis.

**ZL:** Was involved in the writing of the manuscript and is responsible for the integrity of the study.

All authors have read and approved the final manuscript.
